# Optimizing the post-graduate institutional program evaluation process

**DOI:** 10.1186/s12909-016-0586-4

**Published:** 2016-02-17

**Authors:** Monica L. Lypson, Mark E. P. Prince, Steven J. Kasten, Nicholas H. Osborne, Richard H. Cohan, Terry Kowalenko, Paul J. Dougherty, R. Kevin Reynolds, M. Catherine Spires, Jeffrey H. Kozlow, Scott D. Gitlin

**Affiliations:** Veterans Affairs Ann Arbor Healthcare System, 2215 Fuller Road #11J, Ann Arbor, MI 48105 USA; Department of Otorhinolaryngology, University of Michigan Medical School, Ann Arbor, MI USA; Department of Otorhinolaryngology, Ann Arbor, MI USA; University of Michigan Medical School, Ann Arbor, MI USA; Department of Plastic Surgery, University of Michigan Medical School, Ann Arbor, MI USA; Department of Vascular Surgery, University of Michigan Medical School, Ann Arbor, MI USA; Department of Radiology, University of Michigan Medical School, Ann Arbor, MI USA; Department of Emergency Medicine, Beaumont Health System, Royal Oak, MI USA; Detroit Medical Center/Providence Hospital Orthopaedic Surgery Residency Program, Detroit, MI USA; Department of Obstetrics and Gynecology, University of Michigan Medical School, Ann Arbor, MI USA; Physical Medicine & Rehabilitation, University of Michigan Medical School, Ann Arbor, MI USA; Department of Surgery, University of Michigan Medical School, Ann Arbor, MI USA; Department of Internal Medicine, Division of Hematology/Oncology, University of Michigan Medical School, Ann Arbor, MI USA

**Keywords:** Postgraduate medical education, Internal review committee, Quality improvement, Program review

## Abstract

**Background:**

Reviewing program educational efforts is an important component of postgraduate medical education program accreditation. The post-graduate review process has evolved over time to include centralized oversight based on accreditation standards. The institutional review process and the impact on participating faculty are topics not well described in the literature.

**Methods:**

We conducted multiple Plan-Do-Study-Act (PDSA) cycles to identify and implement areas for change to improve productivity in our institutional program review committee. We also conducted one focus group and six in-person interviews with 18 committee members to explore their perspectives on the committee’s evolution. One author (MLL) reviewed the transcripts and performed the initial thematic coding with a PhD level research associate and identified and categorized themes. These themes were confirmed by all participating committee members upon review of a detailed summary. Emergent themes were triangulated with the University of Michigan Medical School’s Admissions Executive Committee (AEC).

**Results:**

We present an overview of adopted new practices to the educational program evaluation process at the University of Michigan Health System that includes standardization of meetings, inclusion of resident members, development of area content experts, solicitation of committed committee members, transition from paper to electronic committee materials, and focus on continuous improvement. Faculty and resident committee members identified multiple improvement areas including the ability to provide high quality reviews of training programs, personal and professional development, and improved feedback from program trainees.

**Conclusions:**

A standing committee that utilizes the expertise of a group of committed faculty members and which includes formal resident membership has significant advantages over ad hoc or other organizational structures for program evaluation committees.

## Background

Calls for the development of a systematic approach to program evaluation in post-graduate medical education (GME) abound [1–3]. Several suggestions to a potential schema include the establishment of institutional tracking systems, dashboards and establishment of quality metrics [1, 4]. The United Kingdom’s General Medical Council, in its development and support of its Foundation Programme, echoes similar sentiments regarding the need to develop mechanisms for curriculum evaluation [5].

Most of the existing literature within the post-graduate milieu has emerged in response to the United States’ accreditor’s (e.g. Accreditation Council for Graduate Medical Education- ACGME) requirements [6]. More specifically, the International Recognition of Excellence in Education’s Areas of Excellence to be Recognized (ASPIRE) program established award criteria in the area of assessment with one of the criteria being to demonstrate “rigorous and continuous quality control process” [7]. This award program is focused on undergraduate medical education; the criteria for the assessment award is based on a program’s ability to assess competence, their commitment to scholarship and innovations as well as their ability to engage learners. ASPIRE’s goals are similar to those listed by the Accreditation Council for Graduate Medical Education’s (ACGME’s) Clinical Learning Environment Review (CLER) Program and the ACGME-International institutional requirements focus on program improvement [6, 8, 9].

As a result of the ACGME’s accreditation standards, continuous review processes for clinical care has been widely accepted within our academic environment [1, 10–12]. The ACGME internal review process has evolved over time to include centralized input and oversight. [13] As part of proposed changes introduced in 2013, the ACGME removed the requirement for internal reviews, yet continued to promote institutional oversight of the annual program review, as well as special reviews as needed. The expertise developed as part of the internal review process will be needed as part of the new review process. These changes in the institutional review process have had positive results. Andolsek et al. recently demonstrated that the internal review process is not only helpful in improving educational programs, but is also key to improving the review teams’ understanding of program requirements and competency teaching [14]. Little has been done to describe the process of the institutional internal review committee as well as the benefits to the participants.

The literature on postgraduate program evaluation is fairly recent and there is little information on how to develop the expertise of committee members who are able and willing to review the large amount of data created by the assessment of competence and quality metrics for post-graduate education. It is in this environment that we sought to answer the following questions: What are the conditions needed for the development of a highly functioning review committee? What can be done with, and beyond, accreditation standards to create an environment for thriving committee participants that see program evaluation for the institution as critical? In that vein, we questioned the University of Michigan Health System’s (UMHS) Internal Review Committee (IRC) on their motivations and career success to try to fully understand their commitment as well as probe the benefit to the institution [15]. Lean thinking was used as the theoretical perspective for our quality improvement process. The core principle of lean thinking is the “endless transformation of waste into value from the customer’s perspective” [16]. One working structure used in lean thinking is the Plan-Do-Study-Act (PDSA) Cycle [16].

In this paper we will describe the decade-long evolution of the Internal Review Committee (IRC) into the Special Program Review Committee (SPRC). This change was made using multiple quality improvement cycles as well as the members’ perspective on the transformation of the committee.

## Methods

To improve the program review process, committee members engaged in multiple PDSA cycles of continuous quality improvement from 2002 to 2013. The PDSA model advocates the formation of a hypothesis for improvement (Plan), a study protocol with collection of data (Do), analysis and interpretation of the results (Study), and the iteration for what to do next (Act) [16]. The improvement process included several training exercises with 12 committee members, using four reports (two ACGME review reports, IRC citation/recommendation report, and an IRC follow-up report) to improve their ability to detect the presence or absence of certain issues. The committee often planned a new activity or intervention to improve their program review accuracy, collect many types of data including informal surveys of our customers (i.e. program directors); study the information and outcomes obtained and then “Act” to enhance their work.

To understand their perspectives on the committee’s evolution, we held a focus group and individual interviews with 18 SPRC members: 11 faculty members, five residents, and two office GME staff. Members of the standing committee included some who had also participated during the ad hoc committee era. The focus group lasted approximately 2.5 h and the in-depth face-to-face interviews lasted 45–90 min each. All participants provided their consent, knowing that they were being recorded.

Focus group data was analyzed using grounded theory methodology, which seeks to understand the manner in which an individual is impacted by a particular experience [17]. One author (MLL) reviewed the transcripts independently and performed the initial thematic coding with a PhD level research associate. Subsequently, they met and reached consensus on the initial themes through discussions and repeated comparison of common themes. As a method of theme validation, the two coders summarized all initial themes and shared them with the study participants to solicit comments and suggestions. These comments were then presented to the initial coders who revisited the transcripts to confirm theme locations and to incorporate them into a final list. When locations were not confirmed, themes were omitted from the final list. As a method of qualitative triangulation, one author (MLL) shared emergent themes with the University of Michigan Medical School’s Admissions Executive Committee (AEC) and asked about their experiences as members of a standing committee. All aspects of this study received exemption status from the University of Michigan Medical School Institutional Review Board.

## Results

Here we provide results of the PDSA process, support from ACGME feedback (via process outcomes compared to Residency Review Committee citations) and data from the focus group suggesting that a standing committee structure has benefit.

### Improvement Cycle 1

Between 1999 and 2001, the institution developed a review process based on, and in compliance with, the ACGME requirements. In 2002–2003 we moved from an ad hoc to a standing committee model specifically convened to review individual training programs. Ad hoc committees would meet the same number of times per year and participants were solicited from over 50 program directors and their associated faculty via email. One hypothesis for this quality improvement project was that a standing committee would provide a comprehensive review and lead to career development opportunities for its members versus those in an ad hoc model.

The intent of the standing committee model was to allow members to gain expertise and familiarity interpreting postgraduate requirements, and evaluating and critiquing training programs. By developing areas of expertise within the review process, members are able to recognize and address patterns of concerns across programs and within the institution. Members also began to learn and promote system improvements to education and they were able to transfer this expertise to the educational operations of their own department’s postgraduate medical training programs.

This standing committee is comprised of 12 faculty (average 8.5 years of service) and 4 residents (one-two year terms) who, over a 14 year period, met 16 to 20 times during each academic year to review an average of 20 programs annually. The committee provides a model for consistency of program evaluation and assessment of compliance with program requirements. With an ad hoc committee, it was difficult for committee members to develop process expertise on various important aspects of accreditation. In addition, an understanding of local best practices can be missed due to variable measurement of compliance.

Committee members are financially rewarded for their participation through their professional development accounts to use for travel, research, or support of junior faculty. The financial reward, which is less than the effort involved, demonstrates that the committee’s work is valued by the institution. As incentives waxed and waned, decreasing over time, the quality of review remained consistent; underlining the faculty’s commitment to the process.

As mentioned above, the membership of the standing committee includes resident members. These members are responsible for coordinating interviews with fellow residents and collecting data on residents’ opinions about their educational program. Resident members serve as active participants on the committee, having a voice equal to that of the committee’s faculty. Inclusion of residents allows for the collection of valuable perspectives of the training programs’ residents, and they are better able (than faculty) to elicit honest feedback from residents who are interviewed, due to the peer-to-peer nature of these interactions.

### Improvement Cycle 2

The move to a standing committee was partially successful. A natural outcome of that was for the committee to begin to use intentional recruiting for membership on the standing committee. Departmental leaders with a sincere interest in the integrity and excellence in the review process were pursued. Faculty for the committee were recruited from training program Assistant/Associate Program Directors and Program Directors, as well as others by recommendation. Residents have the opportunity to participate and are recruited by their peers or are recommended by their program directors. Selection is confirmed by the institution’s Graduate Medical Education Committee.

### Improvement Cycle 3

Due to the high demand of requests and collection of data the committee transitioned from a paper to an electronic format for both the committee meeting materials and program documentation. This change resulted in a substantial cost savings in paper and delivery fees. Prior to this, the review process involved:

- Cumbersome binder process with numerous steps and unnecessary processes.
- High cost due to shelf storage and volume capacity, duplication and delivery of binders
- Excessive effort of postgraduate and department program’s staff in assembling each program’s documentation.

The transition to an electronic process came from requests from Program Directors, Program Coordinators and several committee members. This new streamlined documentation process was the essential element needed for the SPRC to be able to assess the educational components of a training program and for programs to decrease the effort and time devoted to the review process (e.g., collecting and assembling program information and delivering review binders).

### Improvement Cycle 4

The committee had established a well-functioning work flow. However, in 2012 the ACGME changed its accreditation standards as well as noting a need for continuous review at the individual program level as well. In 2013, the IRC became the GME SPRCwith an expanded role for providing oversight of the annual program reviews, reviewing GME special reviews, and performing internal reviews of new programs. Thus, the internal review protocol was modified into a structure that supported a protocol for annual reviews. The items reviewed did not change, however the mechanisms of review became focused on the program’s annual report of their own program evaluation and assessment.

### Improvement Cycle 5

We realized that programs with two 5-year accreditation cycles and no changes in Program Director were preparing the same documentation as programs with fewer years of accreditation and multiple citations. The committee reconfigured the presentation of program materials from a long- to a short-electronic format to reduce the production of unnecessary documentation. After a review of the ACGME’s Next Accreditation System (NAS) requirements, programs with at least two 5-year accreditation cycles were no longer required to submit documentation in areas that they previously demonstrated accreditation compliance. This significantly decreased the volume of documentation received and need for storage, reduced the committee’s review to only key elements needed to assess the educational components of a training program, and saved the committee members and program staff time and effort.

We are currently in the process of implementing a sixth cycle of improvement. Once again, in response to changes in the ACGME requirements there is no longer a need for internal reviews. We have adapted to be able to conduct “impromptu” special reviews for: (1) programs whose annual review submission does not meet the standards of the committee and (2) programs the institution feels are struggling either as a result of lack of resources or issues with learners or leadership. In this model we have moved from a regulatory mandating-based review to an institutional-based annual review program based on program improvement.

### Evidence from ACGME Feedback

The effectiveness of the Special Program Review Committee is supported, in part, by an institutional average accreditation cycle of 4.6 years (2010–2013) prior to the onset of NAS. Under the ad hoc model (2002), the average cycle was approximately 4.2–4.4 years. In addition, formal feedback was provided by Program Directors that the rigor of the review ensured an ‘easy’ ACGME site visit for them. ACGME site visits were less rigorous than their internal review and/or the program was adequately prepared as a result of their internal review. We considered this very helpful given the number of programs and the initial short cycle of new programs. This process has ensured a smooth transition to the NAS for our programs and can serve as a model for other institutions [18]. The NAS established a system of accreditation based on the documentation of educational outcomes, and hopes to reduce the burden of the current structure and process-based approach [18].

### Focus group data

Committee members believed that a standing committee format offered numerous advantages over the ad hoc model.

With the *ad hoc* model there might [be] people that might be doing it (conducting interviews) and this will be the one and only time they’ll ever do it so they won’t get any expertise of what to look for or what standards programs should be held to. (Faculty participant #7)

With this format there is a committee of standing members who are very familiar with reviewing residency documents.

I have, for the last how many years, done the program requirements for each review, so I have read through almost every set of program requirements there are out there and it’s very interesting to see the differences and you start to see the big things that stick out almost every time with any program review you do. For me, it was program evaluation. (Faculty participant #1)

Committee members also commented that the standing committee facilitated the work of program directors, including those who were members of the committee. Because the committee members were aware of common pitfalls and areas for potential improvement, they noted their increased ability to assist program directors in the work they need to do to enhance or further develop their educational programs.

I know that after being on the committee for a while and doing our own internal review…I didn’t try to buff up anything beforehand. I said here’s the program how it exists, tell me what’s wrong, knowing…even if I had there were still going to be changes recommended, why do the work twice. I waited…I was smart…I waited until I had the comments to go on and then made the changes. So I think our committee even goes beyond providing useful information it does some of the work quite frankly for some of these programs and just hands stuff to them. (Faculty participant #1)

Members of the committee frequently brought the expertise that they gained back to their own training programs. In a review of the procedures at Duke University, Andolsek and colleagues also noted that program director involvement in the internal review process often improved the educational content of their own programs [19]. Our findings supported those of Andolsek.

[T]here’s been a huge benefit to my own residents, to the training program, and hopefully to the department as result of what I’ve learned here. (Faculty participant #8)

The standing SPRC structure has demonstrated value over time creating faculty and residents who are skilled and committed to the review process. Committee members reported that the evaluation style of the committee evolved over time, and UMHS program directors were increasingly more likely to view the internal review process as helpful rather than adversarial. As a result, it appears that the SPRC became increasingly more effective as an educational tool for program directors as the committee members gained expertise.

Some of the program directors [are] not all defensive. They see us partnering [with them] much more than when we first started. (Faculty participant #9)

## Discussion

The committee continues to improve upon previous work. In addition to detailed reports, the committee adopted a modified color coded technique for annual program reviews from Duke University. This technique has aided the committee members by quickly identifying different levels of compliance: red (not-compliant), yellow (minimum compliance), green (substantial compliance). The committee has also discussed how to decrease meeting frequency while maintaining efficiency. The committee is adaptable to change but change is challenging at times, even to those who embrace it. It is only through a culture of safety, as well as historical experiences with career and institutional benefits of PDSA cycles, that the committee continues to improve.

The formation of a standing committee with minimal turnover has ensured great stability in the program review process; stability which was absent when ad hoc committees were utilized. This has allowed each member to gain considerable expertise over several years of service which has resulted in increased efficiency and depth of the reviews. Further, this format has led committee members to be able to effectively educate program directors to address deficiencies by exposing them to best practices identified from other training programs within the institution. Consequently, individual program directors have come to view the functions of the committee as beneficial rather than adversarial.

Resident participation on this standing committee has been critical. Resident-to-resident interactions have been very effective in identifying certain problems within reviewed programs. Additionally, resident members place a heavy value on this unique opportunity to learn about the evaluation process, as well as to interact with faculty members as peers.

The program review process is time consuming and requires personal sacrifices; features which are readily admitted by committee members. As a result, the institution has taken the unique step of rewarding committee members for their participation. This monetary reward is appreciated by participating faculty members and is perceived as indicating that the institution understands and values the sacrifices committee members are asked to make. Faculty and resident compensation has also likely contributed to greater stability in the committee membership, even though participating members readily admit that the compensation, in and of itself, is not of sufficient magnitude to lead to their decision to re-enlist year after year. Committee members universally expressed a deep sense of commitment to the educational mission of the institution.

Finally, because committee members serve for multiple years, they are well-positioned to recognize areas in which the internal review process can be improved. This has included facilitating the transition to a paperless process, revising and improving institutional forms utilized in the review process, creating documents which can be adopted by each of the programs (such as examples of different policies), and streamlining the review process. Such improvements would be much more difficult or impossible for an ad hoc committee to identify.

As residency programs move to the NAS, the committee, given its previous quality improvement efforts and ability to adapt quickly to a changing environment, developed a process to provide oversight to 103 programs (as of 2014). This process is electronic, using data already created by programs with a short 15 min questionnaire. The committee uses a red, yellow, green measuring criterion and is able to provide meaningful critiques and meet accreditation requirement in 4–10 meetings a year. Resident participation is continued. This process in the first year will continue to develop as well.

## Conclusion

Under NAS, the institutional GME office will have more responsibility for overseeing its training programs. Although formal site visits will be less frequent for individual training programs than in the past, establishing a standing committee to review programs at regular intervals was part of a logical transition to accomplish this requirement.

## Abbreviation

PDSA: Plan-Do-Study-Act
AEC: Admissions Executive Committee
GME: Post-graduate medical education
ASPIRE: Areas of Excellence to be Recognized
ACGME: Accreditation Council for Graduate Medical Education
CLER: Clinical Learning Environment Review
UMHS: University of Michigan Health System
IRC: Internal Review Committee
SPRC: Special Program Review Committee
NAS: Next Accreditation System
